# Serum Neuron-specific Enolase and S100 Calcium-binding Protein B in Pediatric Diabetic Ketoacidosis

**DOI:** 10.4274/jcrpe.galenos.2019.2018.0280

**Published:** 2019-11-22

**Authors:** Hatem Hamed Elshorbagy, Naglaa Fathy Barseem, Akram Elshafey Elsadek, Ashraf Hamed Al-shokary, Yehia Hamed Abdel Maksoud, Sameh Elsayed Abdulsamea, Iman M. Talaat, Hany Abdelaziz Suliman, Naglaa M. Kamal, Waleed E. Abdelghani, Sanaa Mohammed Azab, Dalia Mohamed Nour El Din

**Affiliations:** 1 Menoufia University Faculty of Medicine, Departments of Pediatrics and Pediatric Neurology, Shebeen Elkom, Egypt; Alhada and Taif Armed forces Hospitals, Departments of Pediatrics and Pediatric Neurology, Taif, Saudi Arabia; 2 Menofia University Faculty of Medicine, Department of Pediatrics, Shebeen Elkom, Egypt; 3 Benha University Faculty of Medicine, Department of Pediatrics, Al Obour, Egypt; 4 Ain Shams University Faculty of Medicine, Department of Pediatrics, Cairo, Egypt; 5 Cairo University Faculty of Medicine, Departments of Pediatrics and Pediatric Hepatology, Cairo, Egypt; Alhada Armed forces Hospital, Clinics of Pediatric Hepatology and Gastroenterology, Taif, Saudi Arabia; 6 Al-Azhar University Faculty of Medicine, Department of Pediatrics, El-Khalifa, Egypt; 7 Benha University Faculty of Medicine, Department of Clinical Pathology, Benha, Egypt

**Keywords:** Neuron-specific enolase, ketoacidosis, brain injury, S100B, type 1 diabetes mellitus, children

## Abstract

**Objective:**

Neuron-specific enolase (NSE) and S100 calcium-binding protein B (S100B) are markers of different neurological disorders. The aim was to investigate the relationship between NSE and S100B serum concentrations and the severity of diabetic ketoacidosis (DKA) in diabetic children.

**Methods:**

Eighty children with DKA, 40 with type 1 diabetes mellitus (T1DM) without DKA and 40 healthy controls were enrolled. Severity of DKA was assessed according to blood pH and bicarbonate concentration. Serum NSE and S100B were measured in all participants. In the DKA group serum NSE and S100B were measured at three time points, at admission and at 12 hours and 24 hours after starting treatment.

**Results:**

Children with DKA showed significantly higher serum levels of NSE at all time points compared to children with T1DM without DKA and controls (p<0.01), while serum S100B concentrations did not differ between the three cohorts. Children with T1DM but without DKA also had significantly higher serum levels of NSE (p<0.01) compared to healthy controls. Patients with low Glasgow Coma Scale score (GCSS) and those with moderate and severe DKA had significantly higher levels of NSE at all time points (p<0.01 for each) compared to patients with normal GCSS and those with mild DKA. No significant differences were found in serum S100B levels according to the severity of DKA and GCS (p>0.05). Younger age, lower GCSS, higher glucose and HbA1c, lower pH and lower serum bicarbonate were the risk factors associated with elevated NSE.

**Conclusion:**

Serum NSE is elevated in all patients with type 1 DM and, in patients with DKA, correlates with severity of DKA. However, serum S100B concentration did not differ between T1DM with or without DKA and healthy controls.

**What is already known on this topic?**

Cerebral edema is the most serious and devastating event in diabetic children during episodes of diabetic ketoacidosis (DKA). However, there are only three studies which have evaluated brain injury markers in children with DKA. These studies report increased plasma levels of neuron-specific enolase (NSE) and S100 calcium-binding protein B (S100B) in patients with DKA.

**What this study adds?**

In contrast to previous reports this study analyzed simultaneous measurement of both NSE and S100 protein B and compared these with children with type 1 diabetes mellitus without DKA and a healthy control group. Serum NSE is elevated in DKA and the increase in NSE concentrations correlate directly with severity of acidosis in DKA. NSE is also significantly elevated in childen with diabetes but without DKA when compared to healthy controls. Thus NSE may be a useful marker of neuronal injury.

## Introduction

Type 1 diabetes mellitus (T1DM) is the most common chronic metabolic disorder in childhood. Twenty-five to forty percent of children with T1DM present with diabetic ketoacidosis (DKA) at the time of diagnosis (1). DKA is the leading cause of morbidity and mortality in children with T1DM. DKA is characterized by a triad of severe hyperglycemia, metabolic acidosis and hyperketonemia (2).

Cerebral edema is the most serious and devastating event during episodes of DKA (3). Fifty four percent of pediatric DKA patients show neurologic injury, which may manifest as headache, dizziness, depressed mood and/or muscle weakness, without overt cerebral edema (2,4). Ghetti et al (2) and others reported that overall cognitive function is affected by even one episode of DKA (5,6). To date, most data on pediatric DKA related to brain injury consist of data derived from animal studies, small observational studies and case reports (7,8). It is known that many risk factors, such as duration and severity of DKA before treatment, intravenous mannitol or hypertonic saline or overhydration, are involved in the development of cerebral edema (9,10,11). Most pediatric patients with DKA suffer an acute neurologic decompensation several hours after the start of treatment, indicating a potential relationship with treatment strategies. The identification of an early marker that can be easily measured in the blood and either precedes or coincides with the clinical decompensation of diabetic children would be of value as an indicator of adjustment of management strategy to minimize neurologic injury (12,13). Recent studies have shown that the estimation of neuronal derived proteins in biologic fluids [serum and cerebrospinal fluid (CSF)] can be used for the evaluation neurologic injury. Hamed et al (14) investigated the utility of the proteins, neuron-specific enolase (NSE), myelin basic protein, S100 calcium-binding protein B (S100B) and glial fibrillary acidic protein (GFAP) in their study. These brain injury biomarkers have been studied in traumatic brain injury, but studies assessing the value of these biomarkers in pediatric DKA are very limited (15,16,17).

In this present study, the aim was to investigate serum concentrations of NSE and S100B and their relationship to different clinical, radiological and laboratory variables in children with DKA and in children with T1DM without DKA and compare these to non-diabetic healthy age-matched controls.

## Methods

This is a cross-sectional case-control study. The patients were recruited from the pediatric outpatient clinic and pediatric intensive care unit of Al Hada and Taif military hospitals, Saudi Arabia. The study included 40 apparently healthy children of matched age and sex who visited the general pediatric outpatient clinic for purposes of immunization and/or routine health monitoring as controls.

Diagnosis of DKA was based on hyperglycemia (>200 mg/dL equivalent to 11.1 mmol/L) and metabolic acidosis (serum pH <7.3, bicarbonate <15 mEq/L equivalent to 15 mmol/L), with evidence of increased ketoacidosis in blood (measurable serum or urine ketones, increased anion gap) (18). The diagnosis of diabetes was confirmed according to the World Health Organization diagnostic criteria (19). Children were subdivided into two groups. Group 1 included children with newly diagnosed T1DM with DKA as a first presentation and group 2 included children with a known diagnosis of T1DM and who developed DKA as a complication. Cerebral edema was diagnosed clinically when patients developed sudden changes in their mental/clinical state, such as a severe headache, recurrent vomiting, seizures, hypertension, inappropriate slowing of the heart rate and/or signs of increased intracranial pressure. Subclinical cerebral edema was defined as minor changes in mental status, with or without being given mannitol therapy, but not developing into overt cerebral edema (3).

Exclusion criteria were:

-  Children less than one year of age.

-  A pre-existing medical condition other than T1DM, such as a neurologic or neurodevelopmental abnormality documented by brain computed tomography or magnetic resonance imaging (MRI).

-  History of recent head trauma.

-  Other known complications of type T1DM (e.g., neuropathy, retinopathy and/or nephropathy).

-  Hypoglycemic attacks.

-  Administration of insulin or intravenous fluids before enrollment.

-  T1DM with a hyperosmolar hyperglycemic state.

**Figure 1:**
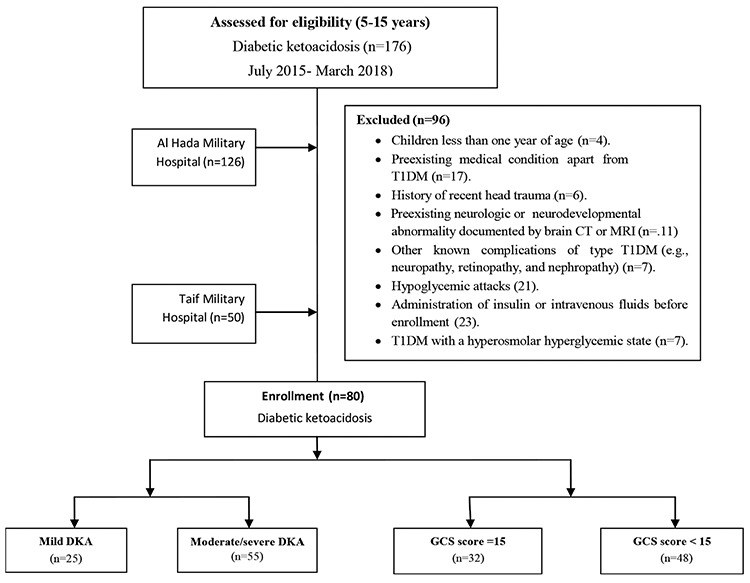
CONSORT flow diagram of patients with diabetic ketoacidosis T1DM: type 1 diabetes mellitus, MRI: magnetic resonance imaging, GCS: Glasgow Coma Scale, DKA: diabetic ketoacidosis

The CONSORT flow diagram of DKA patients is shown in Figure 1.

The study was conducted during the time period from July 2015 to March 2018. Informed consent was taken from all participants in the study. A Local Ethical Committee in Al Hada and Taif military hospitals approved the study (approval number: 53131370). The study protocol conforms to the ethical guidelines of the 1964 Declaration of Helsinki and its later amendments.

All patients were subjected to:

- Complete history taking which included age, gender, age of onset, duration of illness, dose of insulin, insulin regimen, compliance with treatment and history of any episodes of DKA.

- A thorough clinical examination including mental state, assessment of conscious level using Glasgow Coma Scale (GCS) for age and assessment of cranial nerve function (20).

- Laboratory investigations included complete blood count, random blood sugar, serum electrolytes (sodium, potassium, calcium), blood urea, serum creatinine, hemoglobin A1c (HbA1c), osmolality, analysis of arterial blood gases (pH, PO_2_, PCO_2_, and HCO_3_) and analysis of urine for detection of ketone bodies.

DKA ranges from mild to severe and will influence the treatment and disposition of the patient. DKA classification in this study was based on two variables; pH and HCO_3_. Pediatric DKA may be classified as severe (arterial pH <7.1 and HCO_3_ <5), moderate (pH <7.2 and HCO_3_ <10) or mild DKA (pH <7.3 and HCO_3_ <15) (21). According to the GCS score, patients with DKA were divided into (1) patients with GCS score=15 and (2) patients with GCS score <15. All patients with DKA were treated according to the standard guidelines (22). None of our patients received any sedation.

Children with T1DM without DKA were divided into two groups based on metabolic control: (1) patients with good control, with HbA1c values of <7.5; and (2) Patients with poor control with HbA1c values >9.0% (23). There were no children in this group with HbA1c values between 7.5-8.9%. In patients with DKA, a 4 mL venous blood sample was taken at the time of admission for the assay of NSE and S100B concentrations, before the initial saline bolus. Subsequent samples were taken at 12 hours and 24 hours after the start of treatment.

- In the healthy controls and in T1DM patients without DKA, only baseline blood samples were taken.

All blood samples were centrifuged and stored at -80 °C until the time of assay.

NSE and S100B were tested individually by enzyme-linked immunosorbent assays (ELISA) (EMD Millipore, Merck, Germany), according to the instructions of the manufacturer. For the NSE and S100B tests serum volumes of 220 µL and 60 µL were used with sensitivities of 0.19 ng/mL and 50 pg/mL, respectively. The detection limits of the assays were 0-100 ng/mL and 0.25-25 pg/mL for NSE and S100B, respectively (24).

NSE measurements are compromised by even slight hemolysis, as it is abundant in red blood cells. Therefore hemolysis was avoided as far as possible during the procedure. Established pre-analytical precautions were followed to ensure minimal hemolysis and thus greater accuracy of returned results. In those samples which were hemolyzed, a correction was applied and is described briefly hereafter. Toman et al (25) derived an equation to correct for hemolysis in samples by measuring baseline NSE in samples and then intentionally hemolyzing them and remeasuring. The individualized hemolysis correction equation is:

NSE (corrected)=NSE (measured) - [Hb (serum)] [NSE (RBCs/Hb)] + 0.0844 [Hb (serum)] + 1.1.

This was shown to correct 95% of the intentionally hemolyzed samples to within ±5 ng/mL of corresponding baseline NSE concentrations, compared to only 74% of samples using a generalized formula.

This equation was used in the study in an attempt to reduce the sample rejection rates, which approached 11% in our institution.

- Brain imaging: Brain MRI was performed in all patients with DKA, after stabilization and hemodynamic stability, to demonstrate any brain injury.

### Statistical Analysis

Data analysis was carried out using the SPSS software package, version 10.0 (IBM Inc., Chicago, IL, USA). Homogeneity of the data was assessed with the Kolmogorov-Smirnov test. All data sets were normally distributed. Therefore data were presented as means ± standard deviation. ANOVA test was used to test the significance of means. The correlation between different variables was assessed using Pearson correlation test. The determination of risk factors that were significantly associated with increased levels of NSE was performed using multiple logistic regression analysis. The odds ratios and significance at 95% confidence intervals were calculated.

The relationship of NSE with GCS, pH and HCO_3_ was re-evaluated by partial correlation after controlling for age, duration of diabetes and metabolic control (well controlled/poor controlled) using Spearman’s rho test. P<0.05 was considered significant.

## Results

A total of 176 patients with DKA were admitted to the pediatric endocrinology clinic during the study period. Of these patients, 96 were excluded for a number of reasons (see Figure 1). The study was thus conducted with 80 children with DKA and 40 children with T1DM without DKA. The mean age of children with DKA was 10.4±3.6 years and 68.75% of patients were males. The 40 children with T1DM without DKA were 26 (65%) males. Their ages ranged from 4 to15 years with a mean age of 10.7±3.2 years. Demographic and clinical characteristics of patients are shown in (Table 1).

The clinical manifestations of DKA among our patients were rapid acidotic breathing, acetone breath, repeated vomiting, polyuria, polydipsia, enuresis and acute abdomen. Decreased level of consciousness was reported in 60% of children with DKA at the time of admission, indicated by a GCS score <15, suggesting the presence of cerebral edema. Blood pH and serum concentrations of bicarbonate, corrected sodium, urea nitrogen and creatinine were all consistent with the diagnosis of DKA. Mild and moderate DKA was present in 31.25% while severe DKA was reported in 37.5% (Table 1). The proportion of the samples with evidence of hemolysis was 11%, but none exceeded 2%.

Children with DKA showed significantly higher serum concentrations of NSE at the three time points: admission (13.9±2.8 ng/mL); 12 hours (27.8±2.3 ng/mL); and 24 hours (36.7±5.6 ng/mL) after starting treatment compared to children with T1DM without DKA (10.2±2.2 ng/mL, p<0.01) and to healthy controls (5.17±1.5 ng/mL, p<0.01). When NSE concentrations in children with T1DM without DKA were compared with those of healthy controls there were significantly higher concentrations in the T1DM patients (10.2±2.2 ng/mL versus 5.17±1.5 ng/mL, p<0.01, respectively).

No significant difference were found between the studied groups in terms of S100B concentrations (p>0.05). Neither did the serum S100B change significantly at the three time points measured in the DKA group: at admission (53.2±6.7 pg/mL); at 12 hours (52.4±7.2 pg/mL); and 24 hours (50.6±7.7 pg/mL) after starting treatment (p>0.05) (Table 2).

Patients with low GCS score had significantly higher concentrations of NSE at admission (16.7±7.4 ng/mL), 12 hours (30.9±4.8 ng/mL) and 24 hours (22.7±7.1 ng/mL) compared to patients with normal GCS score which were 6.42±2.9 ng/mL, 5.18±2.5 ng/mL and 7.17±0.6 ng/mL at the same time points respectively (p<0.01) and in comparison with T1DM patients without DKA whose mean NSE concentration was 10.2±2.2 ng/mL (p<0.01; see Table 3).

Patients with duration of T1DM ≥5 years had significantly higher mean concentrations of NSE than those with shorter duration, both for patients with DKA (11.17±3.2 ng/mL versus 7.96±2.7 ng/mL, respectively; p=0.038) and for patients without DKA (10.88±3.2 ng/mL versus 6.23±2.3 ng/mL, respectively; p=0.042).

When metabolic control was investigated it was found that serum concentrations of NSE were significantly higher among diabetic children with poor metabolic control without DKA than in those with good control (12.36±3.3 ng/mL versus 6.37±2.4 ng/mL; p=0.032).

Patients with severe DKA had significantly higher mean concentrations of NSE at the each time point compared to patients with moderate DKA (19.6±8.4 ng/mL versus 17.3±7.8 ng/mL; 37.7±5.3 ng/mL versus 33.7±5.3 ng/mL and 28.3±9.3 ng/mL versus 25.3±7.2 ng/mL at admission, 12 and 24 hours, respectively), and compared to patients with mild DKA (19.6±8.4 ng/mL versus 9.12±3.2 ng/mL, 37.7±5.3 ng/mL versus 6.22±2.9 ng/mL and 28.3±9.3 ng/mL versus 9.13±0.9 ng/mL, respectively; p<0.01). The mean NSE concentration was not significantly different from the control group at the 12^th^ hour in the mild DKA group (6.22±2.9 ng/mL versus 5.25±1.2 ng/mL, p>0.05; see Table 4).

There were no differences in mean concentration of S100B between patients with low GCS scores compared with those with normal GCS scores at any of the time points examined (see Table 5). Neither were there differences between the low GCS score group and T1DM patients without DKA at any time point (Table 5). The serum concentration of S100B did not differ between the patients with T1DM with and without DKA when compared for duration of illness (Table 5).

When patients were compared by severity of DKA no significant differences were found in mean concentrations of S100B at any of the time points examined (see Table 6). No significant correlation was found between mean concentration of S100B and any of the patient laboratory or demographic data, regardless of the time point examined.

Serum concentrations of NSE at 24 hours after starting treatment for DKA showed significant negative correlation with age (p=0.0001), GCS score (p=0.0001), pH (p=0.02), and bicarbonate concentration (p=0.04) (see Table 7). However, there was significant positive correlation between mean NSE concentration at 24 hours after starting treatment for DKA and baseline NSE concentration (p=0.0001), duration of illness (p=0.03), random blood sugar concentration (p=0.0001) and HbA1c (p=0.001) (Table 7).

Multiple regression analysis was used to assess the relationship between mean concentration of NSE at 24 hours after starting treatment and a range of risk factors. Significant association was found with age (p=0.001), GCS score (p=0.007), random blood sugar concentration (p=0.008), HbA1c (p=0.03), blood pH (p=0.04) and blood bicarbonate (p=0.003) (see Table 8).

The serum NSE concentration in DKA was further assessed, controlling for covariables that may potentially influence the NSE level, which included age, duration of diabetes and metabolic control status. After adjustment, the serum NSE concentration was still independently associated with GCS, pH and bicarbonate.

MRI of the brain showed no significant abnormalities in any of our patients.

## Discussion

Serum concentrations of NSE and S100B, two markers of neuronal damage, were measured, immediately before and over the course of the first 24 hours of treatment for DKA in pediatric T1DM patients in this study. In addition the relationship between the severity of DKA and markers of brain injury were investigated.

NSE, a soluble protein of 45 kDa, is a glycolytic enzyme present almost exclusively in neurons and neuroendocrine cells, although it is also found in platelets and erythrocytes. Erythrocyte-derived NSE is important when using NSE as a clinical marker of neuronal injury as mild hemolysis of only 2% may increase serum concentrations of NSE five-fold (26). In our subjects the proportion of samples with hemolysis was 11%, but none exceeded the 2% limit. In addition, the correction factor of Toman et al (25) was applied in these samples in order to correct, as accurately as possible, the final NSE serum concentrations prior to comparative analysis.

When neuronal membranes are injured, NSE and S100B will diffuse to the extracellular fluid compartment and to the CSF. Therefore, estimation of these markers in CSF may be a clinically attractive method of assessment, as it may be more sensitive than serum measurement and will largely negate the complication of erythrocyte derived NSE. However, there are additional clinical risks and ethical issues which hinder performing lumbar puncture and CSF collection. Measurement of serum NSE and S100B has been used as evidence of alterations in the blood-brain-barrier (BBB) in certain instances of DKA. Hence, interpretation of results using only serum levels was possible (27).

In the recent literature, there are only three studies which have evaluated brain injury markers in children with DKA. Hamed et al (17) compared serum NSE levels among DKA patients without documented cerebral edema with normal and abnormal GCS scores (GCS <15 and GCS=15) and healthy controls. The results showed that DKA patients with GCS <15 had significantly higher serum NSE concentrations than both the DKA patients with GCS=15 and healthy controls, while the difference between the DKA group with GCS=15 and healthy controls was not significant. These results showed that serum NSE was elevated in DKA and also that it correlated with hyperglycemia, ketosis and acidosis (17). Interestingly children with T1DM without DKA also had significantly higher serum concentrations of NSE compared to healthy controls. The mean NSE concentration did not differ significantly in the mild DKA group at 12 hours after starting treatment when compared to the control group. One explanation for this would be that the severity of acidosis was responsible for the significant increase in NSE.

We did not observe a significant elevation in the level of S100B in the DKA group. This finding can be attributed to a mis-match between the methodology of ELISA kit and the biological characteristics of S100B (28). Unfortunately, ELISA assays take 4-6 hours to run and generally present high inter- and intra-coefficients of variation resulting in a worse functional sensitivity of the assay (29). Coupled with the half-life of S100B being in the range of 60 to 120 minutes in patients with brain injury, the measured net amount of S100B in serum samples will inevitably be less than the original concentration due to the rate of degradation when using ELISA methodology (30).

Çatlı et al (31) (2018) studied NSE, S100B and GFAP levels in 29 patients with DKA, 30 with T1DM and 35 healthy children. They found S100B was significantly higher in the DKA group than the healthy control and T1DM groups, while GFAP and NSE levels were not different from controls and T1DM patients. No significant differences were found in GFAP, NSE and S100B levels according to the severity of DKA, diabetes duration and GCS.

Kaya et al (16) (2015) investigated the pre-treatment and post-treatment oxidant capacity, antioxidant capacity and S100B levels in cases of DKA. They hypothesised that long-term exposure to high blood glucose concentrations leads to an increase in the oxidative stress in patients with DKA that led to an increase in S100B concentration, which implies neuronal damage.

In our study significantly increased serum concentrations of NSE were found in diabetic patients without DKA and without detectable CNS disorders, neuropathies, and retinopathy. In addition, serum concentrations of NSE were significantly higher in diabetic children without DKA but with poor metabolic control than those who showed good control. Hyperglycemia-induced pericyte loss and oxidative stress contribute to BBB disruption (32). These neuroanatomical changes observed in experimental models of diabetes may accurately reflect what is occurring in the clinical setting (33). It was reported that cognitive dysfunction in T2DM appears to be due to perma­nent brain damage with significant elevation in NSE level and correlated with the level of glycemic control (34).

Gonder-Frederick et al (35) (2009) reported a disturbance in the cognitive functions of school-aged children with T1DM due to repeated attacks of hyperglycemia. A previous study, conducted by Antenor-Dorsey et al (36), observed changes in brain imaging in the form of increased diffusiveness in the superior parietal lobule and hippocampus attributed to repeated attacks of hyperglycemia, associated with ketosis with or without academia. Experimental and human studies have indicated that chronic hyperglycemia associated with DM resulted in a brain injury which particularly affected memory and learning abilities. The mechanisms underlying brain injury in experimental models include: disruption of BBB; alteration of insulin transporter and decrease in insulin receptors, which are expressed in discrete neuronal populations in the CNS; reduction in the uptake of glucose into the neurons; impairment of energy metabolism; and impairment of the capacity of the brain to generate the connections vital to memory and learning (37). Other investigators reported raised concentrations of NSE in diabetic patients with and without overt neurologic complications (38). It is well-known that T1DM has long-term complications affecting cognitive functions (39). An understanding of the nature and onset of the neurological insults associated with diabetic children is essential to prevent or mitigate these complications.

Among diabetic patients, high blood glucose has been associated with elevated concentrations of serum NSE. In addition to central nervous system disorders, hyperglycemia-induced pericyte loss contributes to disruption of BBB (32).

Another important finding of our study was the elevation in serum concentrations of NSE during DKA and its correction after starting treatment. Our findings support the hypothesis that during the critical time-period where acute complications of DKA have been reported, the levels of NSE remain high (17). In our study, we reported significantly higher serum concentrations of NSE in patients with GCS score <15 compared to patients with normal GCS score at each time point. We also found significantly higher serum concentrations of NSE in patients with moderate to severe DKA compared to patients with mild DKA at the same three time points. It was notable that the concentrations of NSE remained high and were continuing to rise at 24 hours post start of treatment, coinciding with the initial recovery of clinical manifestations of DKA. The persistence of higher concentrations of NSE after improvement of the manifestations of DKA suggests that neuronal injury may recover partially but not completely. Alternatively, the healing process in neurological tissues is know to be relatively slow and so persistent NSE elevation after the first 24 hours of DKA might be expected. It was notable that T1DM children without overt DKA also had significantly higher concentrations of NSE (p<0.01) than non-diabetic children, which also supports the hypothesis that neuronal injury in T1DM may be permanent. An alternative hypothesis would be that neuronal injury may begin early at the cellular level in the context of T1DM without DKA and may be associated with cognitive impairment. Repeated episodes of DKA may carry the risk of progressive neuronal injury. Also, we observed some patients with normal GCS score had a significant elevation in the serum concentrations of NSE after improvement of their condition. This suggests neuronal injury may occur in the absence of brain edema in children with DKA. Our data were consistent with previous studies that reported evidence of neuronal injury without brain edema. Wootton-Gorges et al (40) reported a progressive decrease in N-acetyl aspartate/creatine ratio as evidence of permanent brain injury in a teenager with T1DM and repeated episodes of DKA without clinically apparent cerebral edema.

S100B is a relatively small protein, 9-14 kDa, synthesized largely by glial cells although a small proportion is synthesized by neurons, Schwann cells and in non-neuronal peripheral sources, including cardiomyocytes, alveolar cells, chondrocytes and adipocytes (41).

In our study, S100B protein did not show significant differences between the DKA, T1DM, and healthy control groups although this may have been due to methodological problems, as described earlier.

In research studies, statistical power is generally calculated with two main objectives. Firstly, it can be calculated before data collection to decide the sample size needed for the current study based on information from previous studies. Secondly, it can also be calculated after data analysis. When the result turns out to be non-significant, statistical power can be calculated to verify whether the non-significance result is due to lack of relationship between the groups or due to the lack of statistical power (42). The power of our study was calculated for the comparison of NSE and S100B between children with DKA, children with T1DM without DKA and controls using G power software version 3.1.2. 9. We took into consideration the mean values of NSE and S100B in the studied groups and the Alpha level was kept at 0.05. The power calculated was 1.0 and 0.845 for NSE and S100B respectively. We can confirm that the non-significant result of the S100B analysis between the three groups is robust, as no difference was detected although there was sufficient power (0.845) to detect any difference, if present.

There are a few controversial studies showing that S100B can be used as a marker for cerebral edema in pediatric DKA (15,16,43). Experimental studies reported low levels of S100B in the DKA group. This finding was attributed to glial cell dysfunction and not glial cell loss and that S100B is not a reliable marker of neuronal injury (15). We did not detect any significant increase or decrease in S100B in children with DKA over a short term follow-up (12 hr-24 hr) to predict neuronal injury. We did not find any correlation between S100B concentrations and other laboratory and demographic factors at the time points studied. It was reported that S100B levels were not raised in subclinical cerebral edema in children with DKA (31). However, Kaya et al (16) found significantly higher S100B concentrations in children with DKA but without accompanying cerebral edema than controls, but did not find a significant difference in S100B concentrations before and after initiation of therapy.

In our study, we found the risk factors and early predictors of higher serum concentrations of NSE in children with DKA were younger age, lower GCS score, higher degrees of hyperglycemia, longer duration of illness and more severe acidosis and ketosis. Previous studies have shown that a range of factors are suitable predictors of higher levels of NSE during DKA, but none of these variables has been singled out as the most important determining factor (17,44,45). Regarding clinical risk factors, the degree of acidosis and younger age appeared to be the greatest risk factors for alterations in cerebral structure. However, the degree of acidosis was the most important determining factor of an impaired level of consciousness in children with DKA without cerebral edema (46). Different biomarkers reflecting inflammation including tumor necrosis factor-alpha and interleukin-6 and cerebral dysfunction and/or possible injury (S100B, GFAP), as well as genetic markers of brain injury risk in children with DKA, were studied by Nett et al (47) They demonstrated the potential importance of these markers in the pathophysiology of CNS dysfunction and/or possible injury in DKA.

Under normal conditions with an intact BBB, brain-derived proteins of different molecular weights (such as S100B and NSE) do not cross the BBB (24,46,47). With the disruption of the BBB, blood levels of these proteins can be used as a marker for brain injury (48). During the treatment of DKA, it was observed that the whole brain and regional BBB permeability increased in most patients (33). Although the mechanisms underlying the increase in BBB permeability is still unclear, it is suggested that DKA can disrupt the tight endothelial junctions through inflammatory and immunologic responses (49). Furthermore, many factors such as matrix metalloproteinase activity, hyperglycemia and insulin administration are associated with increased permeability of the BBB (33).

### Study Limitations

We did not measure NSE in the CSF of our patients for as our scientific committee did not approve CSF sampling for the study although CSF measurements would likely be more sensitive to CNS damage. We did not repeat MRI to detect subclinical cerebral edema at the time of diagnosis and during clinical follow-up. Also, neurocognitive function, which is a good marker of brain dysfunction, was not assessed. This is explained by the fact that our study focused on investigating serum concentrations of NSE and S100B in children with DKA and its relationship with different clinical and laboratory variables. The study only examined serum concentrations of NSE and S100B over a period of 24 hours. Longer follow-up would have clarified the progression of serum NSE concentrations as they returned to normal. Lastly, we did not undertake a sample size calculation before conducting our study.

## Conclusions

Serum NSE was found at a significantly higher concentration in T1DM children, with or without DKA, than non-diabetic children. This might suggest a degree of neurologic dysfunction, even in the absence of DKA. In our study, cerebral edema was absent in brain imaging in children with DKA. Elevated NSE concentrations in patients with abnormal GCS and the positive correlation between NSE and severity of acidosis suggest that NSE might be a reliable marker of neuronal injury. However, S100B did not show a simultaneous increase with NSE. This can be attributed to a methodological error with the ELISA kit. To clarify subclinical brain injury related to pediatric DKA, further longer term and larger studies are recommended to assess neurocognitive functions.

## Figures and Tables

**Table 1:**
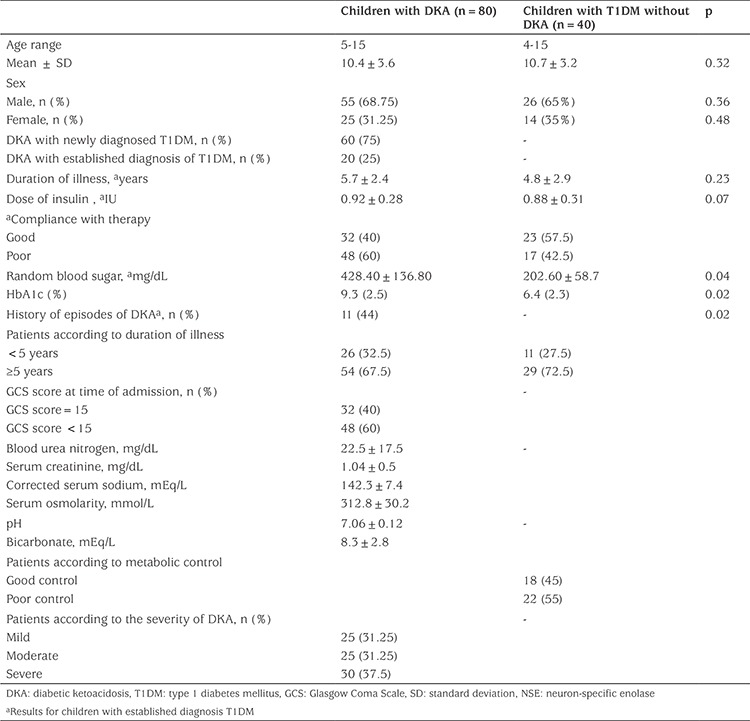
Clinical, demographic and laboratory data of the studied patients

**Table 2:**
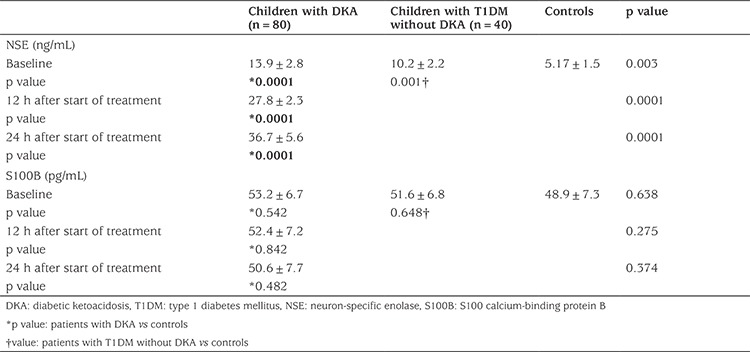
Neuron-specific enolase and S100 calcium-binding protein B at 3-time points among studied groups

**Table 3:**
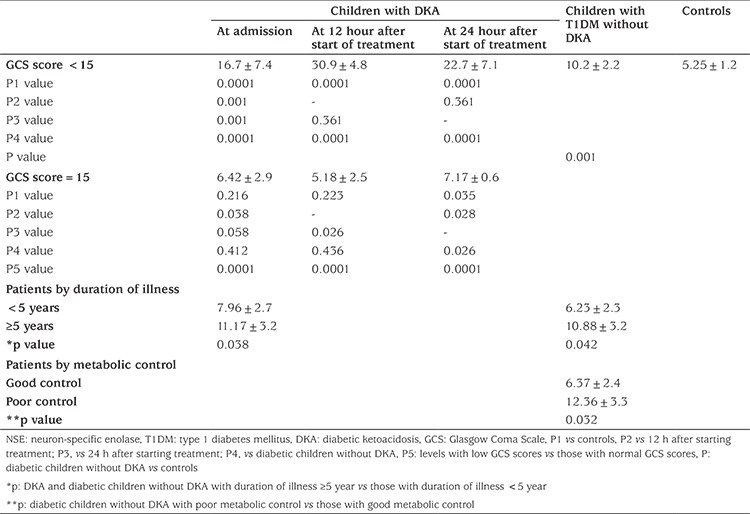
Serum levels of neuron-specific enolase (ng/mL) by Glasgow Coma Scale, duration of illness and metabolic control

**Table 4:**
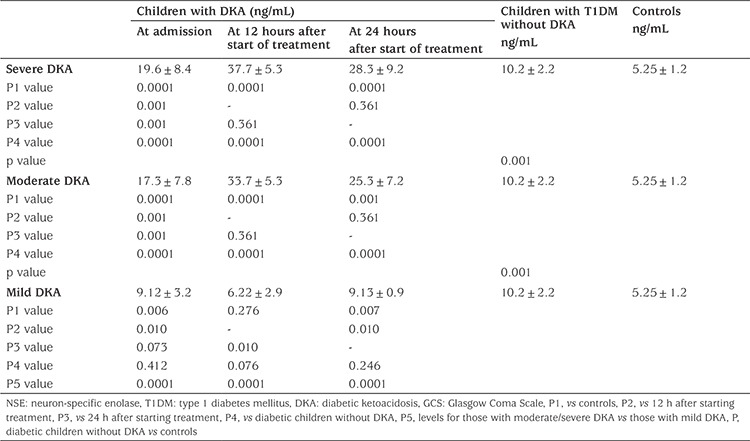
Serum levels of neuron-specific enolase (ng/mL) of the subjects by severity of diabetic ketoacidosis

**Table 5:**
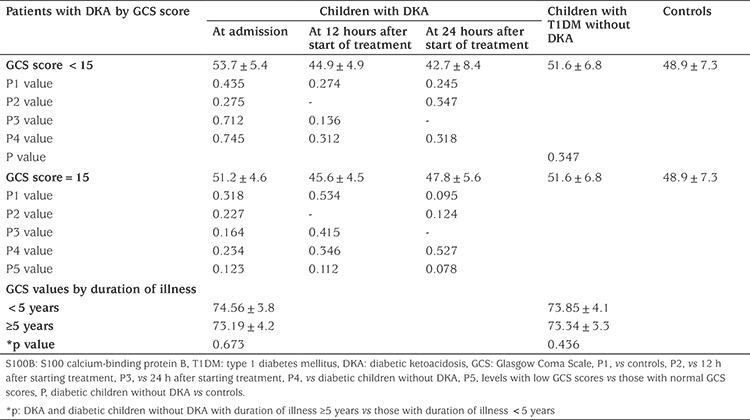
Serum levels of S100 calcium-binding protein B (pg/mL) of the subjects by Glasgow Coma Scale and duration of illness

**Table 6:**
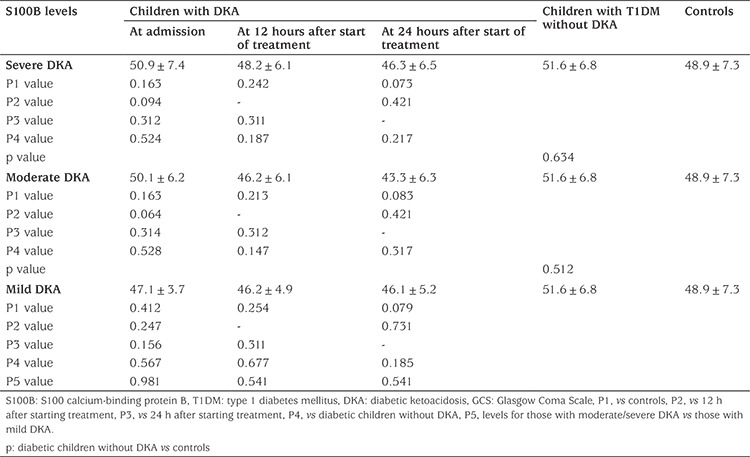
Serum levels of S100 calcium-binding protein B (pg/mL) by severity of diabetic ketoacidosis

**Table 7:**
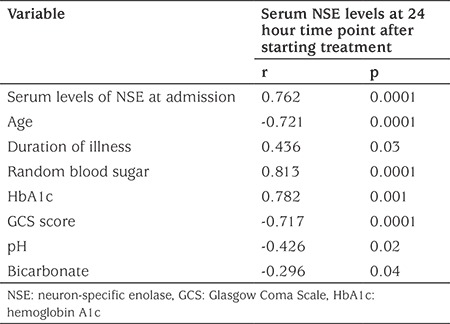
Correlations between the serum levels of neuron-specific enolase and clinical, demographic and laboratory variables among diabetic children with diabetic ketoacidosis

**Table 8:**
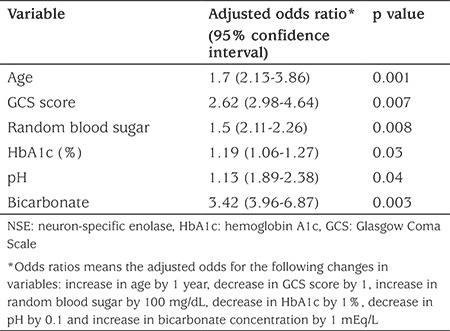
Multiple regression analysis of risk factors associated with increased serum levels of neuron-specific enolase in diabetic children with diabetic ketoacidosis
